# A critical role for suppressors of cytokine signaling 3 in regulating LPS-induced transcriptional activation of matrix metalloproteinase-13 in osteoblasts

**DOI:** 10.7717/peerj.51

**Published:** 2013-03-05

**Authors:** Anqi Gao, Alpdogan Kantarci, Bruno Schneider Herrera, Hongwei Gao, Thomas E. Van Dyke

**Affiliations:** 1 Department of Periodontology, The Forsyth Institute, Cambridge, MA, United States; 2 Center for Experimental Therapeutics and Reperfusion Injury, Department of Anesthesiology, Perioperative & Pain Medicine, Brigham and Women’s Hospital, Harvard Medical School, Boston, MA, United States

**Keywords:** Inflammation, Periodontitis, Cytokine, Osteoblasts

## Abstract

Suppressor of cytokine signaling 3 (SOCS3) is a key regulator of cytokine signaling in macrophages and T cells. Although SOCS3 seems to contribute to the balance between the pro-inflammatory actions of IL-6 family of cytokines and anti-inflammatory signaling of IL-10 by negatively regulating gp130/Jak/Stat3 signal transduction, how and the molecular mechanisms whereby SOCS3 controls the downstream impact of TLR4 are largely unknown and current data are controversial. Furthermore, very little is known regarding SOCS3 function in cells other than myeloid cells and T cells. Our previous study demonstrates that SOCS3 is expressed in osteoblasts and functions as a critical inhibitor of LPS-induced IL-6 expression. However, the function of SOCS3 in osteoblasts remains largely unknown. In the current study, we report for the first time that LPS stimulation of osteoblasts induces the transcriptional activation of matrix metalloproteinase (MMP)-13, a central regulator of bone resorption. Importantly, we demonstrate that SOCS3 overexpression leads to a significant decrease of LPS-induced MMP-13 expression in both primary murine calvariae osteoblasts and a mouse osteoblast-like cell line, MC3T3-E1. Our findings implicate SOCS3 as an important regulatory mediator in bone inflammatory diseases by targeting MMP-13.

## Introduction

Prolonged inflammation is often the reason behind substantial bone loss. In fact, bacteria-induced inflammation is the major cause of bone loss in many bone diseases such as periodontitis, septic arthritis, and osteomyelitis (Nair et al., 1996; Yip et al., 2004). MMP-13 is a member of the matrix metalloproteinase family, a group of structurally and functionally related enzymes responsible for the proteolytic degradation of extracellular matrix components including collagen fibrils in the bone matrix (Massova et al., 1998; Woessner, 1991). Among the MMPs expressed in osteoblasts, MMP-13 is predominantly up-regulated by systemic bone resorbing factors such as parathyroid hormone (Quinn et al., 1990). There is little to no expression of MMP-13 in normal adult tissue, as the enzyme is primarily expressed in hypertrophic chondrocytes, periosteal cells, and osteoblasts during human fetal development and re-expressed in diseases which require tissue repair and remodeling (Tardif et al., 2004). This specific activity of MMP-13, together with its ability to degrade both type I collagen (the primary extracellular matrix component secreted by osteoblasts in the trabecular bone) and type II collagen (the primary fibrillar extracellular matrix component secreted by resting and proliferating chondrocytes in the growth plate) (Inada et al., 2004; Stickens et al., 2004; Tardif et al., 2004) suggests it to be a central agonist of bone resorption and an important target in inflammatory bone diseases. Supporting this hypothesis, lack of MMP-13-mediated type I collagen degradation could explain the increased trabecular bone volume in MMP-13 KO mice (Inada et al., 2004; Stickens et al., 2004; Tardif et al., 2004). On the other hand, increasing evidence suggest that bone-building osteoblasts stand at the interface between bone turnover and innate immunity. It has been reported that lipopolysaccharide (LPS) from *Escherichia coli* (*E. coli*) bacteria up-regulates the expression of several pro-inflammatory mediators in osteoblasts (Nair et al., 1996; Yan et al., 2010), but it is not known whether LPS can induce MMP-13 gene expression in osteoblasts. Given the extensive degradation activity of MMP-13 and its increased presence in inflammatory bone diseases, a better understanding of MMP-13 expression and regulation may lead to therapeutic strategies aimed at inhibiting bone destruction.

SOCS3 is a SOCS box-containing molecule that inhibits signal transducer and activator of transcription (STAT)/Janus kinase (JAK) signaling (Babon & Nicola, 2012). The expression and function of SOCS3 have been investigated mainly in immune cells including macrophages and T cells. Particularly, SOCS3 expression in T cells has been shown to regulate onset and maintenance of allergic responses (Seki et al., 2003). Likewise, a recent study shows that SOCS3 in macrophages negatively regulates neuroinflammatory responses (Qin et al., 2012). Besides STAT/JAK-dependent cytokines, SOCS3 expression can also be induced by a variety of other stimuli including TLR ligands (Heeg & Dalpke, 2003). In fact, SOCS3 is one of the most abundantly induced proteins by LPS in macrophages (Yoshimura, 2009). However, detailed mechanisms by which SOCS3 regulates signaling pathways distinct from STAT/JAK are still largely unknown.

Expression and function of SOCS3 in bone have also been studied, but investigations remain in infant stages. Previous studies show that over-expression of SOCS3 suppresses both acute inflammation induced by staphylococcal enterotoxin B/LPS and inflammatory arthritis induced by interleukin-1β (IL-1β) or collagen (Shouda et al., 2001; Veenbergen et al., 2008; Wong et al., 2006). However, due to the embryonic lethality of SOCS3 knockout mice (Roberts et al., 2001), the role of SOCS3 in inflammatory bone diseases remains to be determined. Further, little data is available for the expression and function of SOCS3 in osteoblasts. Based on our recent study that over-expression of SOCS3 inhibits LPS-induced IL-6 production in osteoblasts (Yan et al., 2010), it is possible that SOCS3 could down-regulate other pro-inflammatory mediators induced by LPS in osteoblasts and therefore play a key role in osteoblast-mediated immune signaling. In this report, we show that LPS stimulation induces a dramatic increase of MMP-13 mRNA expression in both primary murine calvariae osteoblasts and mouse osteoblast-like cells, MC3T3-E1. Importantly, our findings implicate a novel role for SOCS3 in the suppression of LPS-induced MMP-13 transcription in osteoblasts.

## Materials & Methods

### Cell line and reagents

Osteoblast-like MC3T3-E1 cells (American Type Culture Collection, Manassas, VA) were cultured in α-MEM media supplemented with 10% fetal calf serum (FBS) and 1% Penicillin/Streptomycin (P/S), and maintained in a humidified incubator at 37 °C with 5% CO_2_. LPS from *Escherichia coli* (serotype 0111:B4, Sigma, St. Louis, MO) was obtained from Sigma. p38 MAP kinase inhibitor VIII was purchased from EMD Biosciences (Gibbstown, NJ) and dissolved in DMSO for a stock concentration of 50 µM. When used for cell treatment, a 1:5000 dilution was made to achieve a final working concentration of 10 µM. For vehicle control, same dilution of DMSO (1:5000) was performed and then added to the medium.

### Isolation and culture of primary calvarial osteoblasts

Neonatal mice calvarial osteoblasts were isolated from 3 days old mouse litters by dissection of the scalp skin and removal of the calvaria as described in a previous publication (Palmqvist et al., 2002). Calvaria were first incubated in α-MEM media supplemented with 10% fetal bovine serum (FBS), 10,000 IU penicillin and 10,000 ug/mL streptomycin, and then incubated for 10 min in 4 mM Na_2_EDTA solution 3 times; the supernatants were discarded. The calvaria were then incubated twice at 37 °C with 5% CO_2_ in a freshly prepared collagenase type 2 solution containing NaCl, KCl, and NaH_2_PO_4_ X H_2_O on an orbital shaker; the supernatants were discarded. Then, the digests from five sequential incubations with collagenase were pooled, centrifuged, and resuspended in α-MEM media supplemented with 10% FBS, 10,000 IU penicillin, and 10,000 ug/mL streptomycin. Cells were plated in 75 ml flasks. When 90% confluency was reached, the cells were passaged into 24 well-plates at a density of 4 × 10^4^ cells/well.

### RNA isolation and quantitative real time-polymerase chain reaction (qRT-PCR) analysis

Total cellular RNA were extracted from cells with TRIzol (Invitrogen, Carlsbad, CA). RNA concentration was determined by Nanodrop spectrophotometer (Wilmington, DE). First-strand cDNA was synthesized from 2 µg and 1 µg total RNA from MC3T3-E1 and primary cells, respectively, using the Superscript II RNase H^−^ Reverse Transcriptase (Invitrogen, Carlsbad, CA). The data were normalized against glyceraldehyde 3-phosphate dehydrogenase (GAPDH), which was used as a loading control. PCR was performed with primers for SOCS3: 5^′^ primer, 5^′^-AGCTCCAAAAGCGAGTACCA- 3^′^ and 3^′^ primer, 5^′^-TGACGCTCAACGTGAAGAAG- 3^′^; MMP-13: 5^′^ primer, 5^′^-TGGAGTGCCTGATGTGGGTGAATA- 3^′^ and 3^′^ primer, 5^′^-TGGTGTCACATCAGACCAGACCTT- 3^′^; GAPDH: 5^′^ primer, 5^′^-TGCACCACCAACTGCTTAG- 3^′^ and 3^′^ primer, 5^′^-GGATGCAGGGATGATGTTC- 3^′^. The fluorescence of the accumulated double-stranded products was monitored in real time. Gene expression was determined using the equation: “relative expression” = 2^(−ΔCt)^, where ΔCt = Ct_gene of interest_ − Ct_ctrl_.

### Adenovirus transfection

Construction, amplification, and titering of recombinant adenovirus containing mouse SOCS3 or siRNA for SOCS3 have been described previously (Yan et al., 2010). Briefly, full-length mouse SOCS3 cDNA was first inserted into pDNR-CMV donor vector resulting in the production of pDNR-CMV-SOCS3, which has loxP sites both upstream and downstream of the SOCS3 expression cassette. SOCS3 expression cassette was then transferred from pDNR-CMV-SOCS3 to pLP-Adeno-X-CMV viral DNA using Cre-loxP recombination (BD Biosciences, Palo Alto, CA), resulting in Adeno-SOCS3. To generate the virus, Adeno-SOCS3 was digested with PacI and transfected to HEK-293 cells according to the manufacturer’s instructions. Adenoviral siRNA for SOCS3 (Adeno-sh-SOCS3) was generated by Welgen under the control of the cytomegalovirus promoter. Recombinant adenoviruses were purified by BD Adeno-X virus purification kit (BD Biosciences, Palo Alto, CA). The viral stocks were tittered using Adeno-X Rapid Titer Kit (D Biosciences, Palo Alto, CA). MC3T3-E1 cells were grown to 90% confluence and infected with adenovirus (Adeno-Ctrl, Adeno-SOCS3, or Adeno-sh-SOCS3) at 100 multiplicity of infection (MOI). Cells were harvested for gene expression analysis after 48 h of transfection. In some groups, the cells were treated with LPS for 6 h prior to harvesting. Total RNA were isolated and reverse-transcribed into single-stranded cDNA for qRT-PCR analysis. In another experiment, MC3T3-E1 cells were treated with LPS for 0, 15, 30, and 60 min, respectively, prior to harvesting. Whole cell proteins were isolated for western blot analysis.

### Western blot analysis

The cells were lysed in ice-cold radioimmunoprecipitation (RIPA) buffer (Fisher Scientific, Rockford, IL). Following protein extraction, total 80 µg of proteins were used to perform electrophoresis in a 12% polyacrylamide gel and then transferred to a polyvinylidene fluoride (PVDF) membrane at 25 V for 60 min. Membranes were incubated with rabbit anti-SOCS3 antibody (Abcam, Cambridge, MA, 1:1000), rabbit anti-p38 antibody (Cell Signaling Technology, Danvers, MA, 1:1000), or rabbit anti-p-p38 antibody (Cell Signaling Technology, Danvers, MA, 1:1000), respectively at 4 °C for overnight. After 3 sequential washes with TBS-T (0.1% Tween), the membranes were incubated with horseradish peroxidase (HRP)-conjugated mouse anti-rabbit IgG (Fisher Scientific, Rockford, IL, 1:3000) for 1 h at room temperature. The membranes were developed by enhanced chemiluminescence technique (Pierce, Indianapolis, IN).

### Luciferase assay

MC3T3-E1 cells were grown to 60–80% confluence and then transfected with indicated DNA plasmids (0.5 µg total) using Fugene 6 Transfection Reagent (Roche, Indianapolis, IN) as instructed. Twenty-four hours after transfection, the cells were either incubated with or without LPS for 4 h. Cell lysates were then collected and analyzed for luciferase activity by using the Dual-Luciferase Reporter Assay System (Promega, Madison, WI).

### Statistical analysis

All data were analyzed using a One-Way ANOVA, and individual group means were then compared with a Newman-Keuls test.

## Results

### LPS actions on MMP-13 and SOCS3 gene expression in osteoblasts

**Figure 1:**
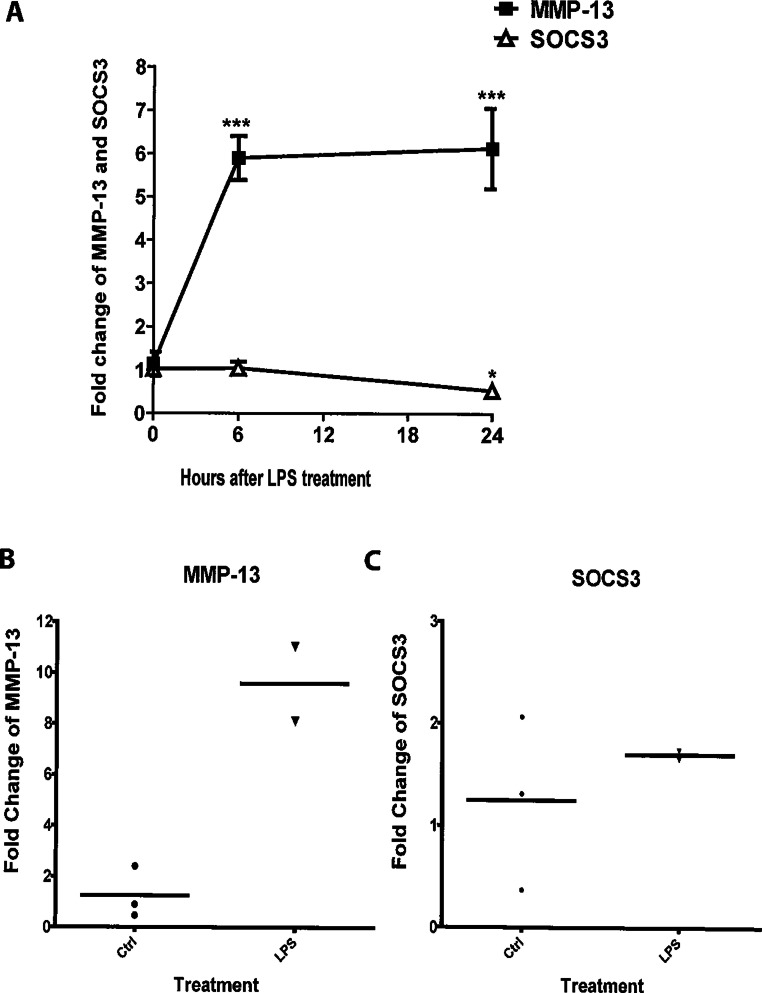
LPS induces expression of MMP-13 and SOCS3 in MC3T3-E1 cells and primary calvarial osteoblasts. MC3T3-E1 cells (A) and primary calvarial osteoblasts (B and C) were incubated with 100 ng LPS/ml for indicated times. Total cellular RNA was isolated. Oligonucleotide primers used for real time PCR were MMP-13, SOCS3 and GAPDH. For panel A, data are presented as mean ± S. E. M., *n* = 4–5, and a representative experiment of a total of two similar experiments is shown. ∗ and ∗∗∗ indicate a statistically significant difference- *p* < 0.05 and *p* < 0.001, respectively.

To assess changes in MMP-13 and SOCS3 gene expression during osteoblast inflammatory response, MC3T3-E1 cells were stimulated with LPS for 0, 6, and 24 h; qRT-PCR results indicated that cells stimulated with LPS for 6 and 24 h exhibited a significant increase (∼six folds for each time point) of MMP-13 gene expression in comparison with non-stimulated cells (Fig. 1A). Conversely, SOCS3 gene expression was significantly reduced 24 h after LPS stimulation (two folds) (Fig. 1A). In addition, primary calvarial osteoblasts showed an ∼eight-fold increase in MMP-13 gene expression after stimulation with LPS for 24 h (Fig. 1B); however, LPS had modest impacts on SOCS3 expression (Fig. 1C).

### SOCS3 impact on LPS-induced MMP-13 gene expression in osteoblasts

**Figure 2:**
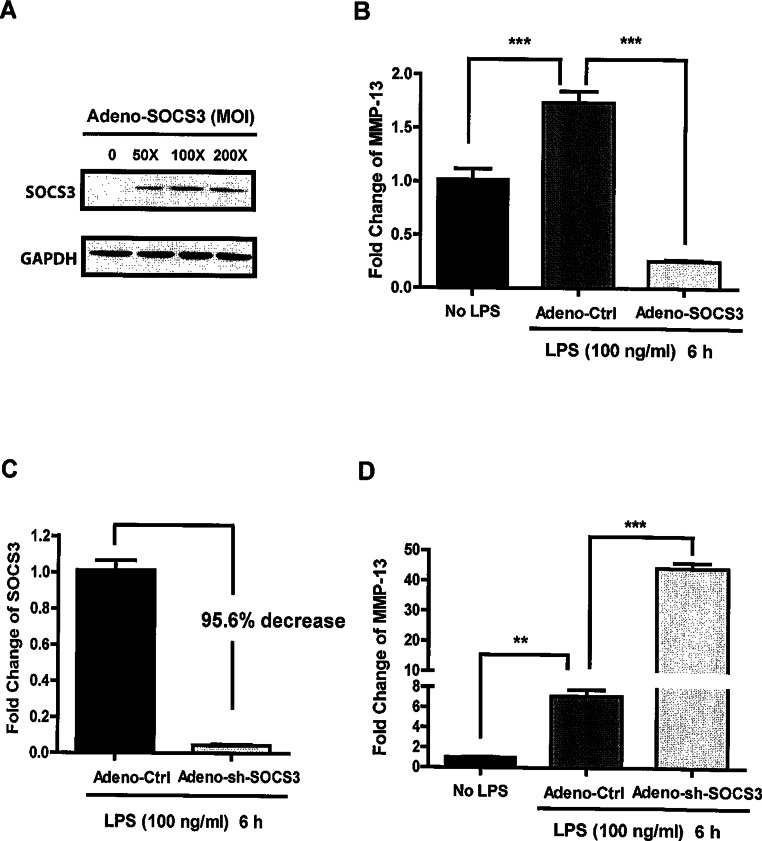
Critical role of SOCS3 in regulating LPS-induced MMP-13 gene expression in MC3T3-E1 cells. (A) MC3T3-E1 cells were infected with Adeno-SOCS3 at indicated MOI. 48 h after infection, the total protein extracts were subjected to Western blot using antibodies against SOCS3. (B) MC3T3-E1 cells were infected with Adeno-Ctrl or Adeno-SOCS3 at 100 X MOI. 48 h later, the cells were stimulated either with or without 100 ng LPS/ml for 6 h. Total cellular RNA was isolated. Oligonucleotide primers used for real time PCR were MMP-13 and GAPDH. Data are presented as mean ± S. E. M., *n* = 4–6. A representative experiment of a total of three similar experiments is shown. ∗∗∗ indicates a statistically significant difference-*p* < 0.001. (C) MC3T3-E1 cells were infected with Adeno-Ctrl or Adeno-sh-SOCS3 at a MOI of 100. 48 h after infection, the cells were incubated with 100 ng LPS/ml for 6 h. RNAs were isolated. Oligonucleotide primers used for real time PCR were SOCS3 and GAPDH. (D) MC3T3-E1 cells were infected with Adeno-Ctrl or Adeno-sh-SOCS3 at a MOI of 100. 48 h later, cells were incubated with or without 100 ng LPS/ml for for 6 h. RNAs were isolated. Oligonucleotide primers used for real time PCR were MMP-13 and GAPDH. Data are presented as mean ± S. E. M., *n* = 4–7. A representative experiment of a total of two similar experiments is shown. ∗∗ and ∗∗∗ indicate a statistically significant difference- *p* < 0.01 and *p* < 0.001, respectively.

We first show that over-expression of SOCS3 via transfection with various MOI (50X, 100X, and 200X) adenoviruses carrying the SOCS3 gene causes a significant increase in SOCS3 protein levels in MC3T3-E1 cells (Fig. 2A). Next, we determined whether SOCS3 expression (100X MOI) in osteoblasts has any impact on LPS-induced MMP-13 expression. As shown in Fig. 2B, MC3T3-E1 cells stimulated with LPS in the presence of SOCS3 protein exhibited a significant decrease (85%, *p* < 0.001) in MMP-13 gene expression levels when compared with cells treated with LPS, but transfected with control viruses. In addition, over-expression of SOCS3 also led to a significant decrease of basal MMP-13 expression (74%, *p* < 0.001) (Fig. 2B). We evaluate whether SOCS3 knockdown has any effect on LPS-induced MMP-13 expression. We first demonstrate that adenovirus-mediated shRNA for SOCS3 (Adeno-sh-SOCS3) led to a significant reduction of the endogenous SOCS3 expression in MC3T3-E1 cells (>95% reduction compared with control virus-treated cells) (Fig. 2C). We next show that adeno-sh-SOCS3 infection of MC3T3-E1 cells resulted in a significant augmentation of MMP-13 gene expression induced by LPS stimulation when compared with cells infected with control virus (3.8 fold induction vs 53 fold induction, *p* < 0.001) (Fig. 2D). These results together implicate an inhibitory role for SOCS3 in LPS-induced MMP-13 gene expression in osteoblasts.

**Figure 3:**
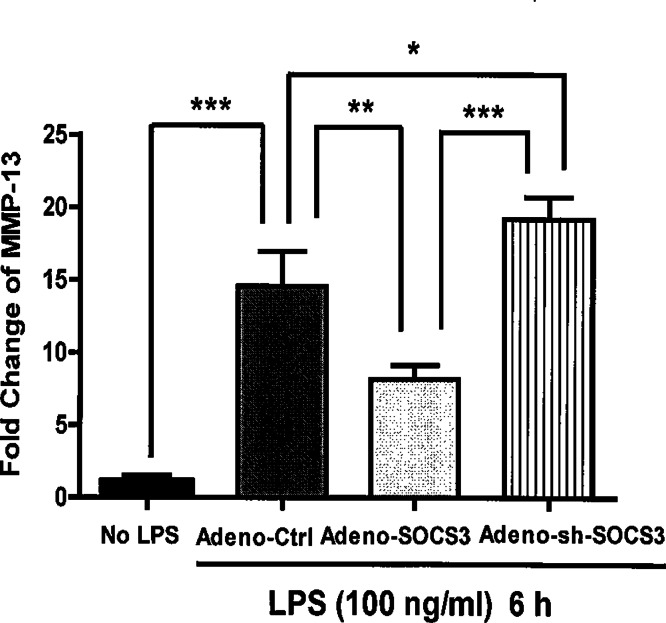
Critical role of SOCS3 in regulating LPS-induced MMP-13 gene expression in primary calvarial osteoblasts. Primary calvarial osteoblasts were infected with Adeno-Ctrl, Adeno-SOCS3, or Adeno-sh-SOCS3 at 100 X MOI. 48 h later, the cells were stimulated either with or without 100 ng LPS/ml for 6 h. Total cellular RNA was isolated. Oligonucleotide primers used for real time PCR were MMP-13 and GAPDH. Data are presented as mean ± S. E. M., *n* = 6. ∗, ∗∗, and ∗∗∗ indicate a statistically significant difference-*p* < 0.05, *p* < 0.01, and *p* < 0.001, respectively.

We next examined the ability of SOCS3 inhibition on MMP-13 expression in primary calvarial osteoblasts. Consistent with the results from MC3T3-E1 cells, LPS treatment of primary calvarial osteoblasts significantly induced MMP-13 gene expression (*p* < 0.001) (Fig. 3). Importantly, adenovirus-mediated SOCS3 over-expression in primary calvarial osteoblasts led to a significant reduction of MMP-13 expression (44%, *p* < 0.01) (Fig. 3). In addition, adeno-sh-SOCS3 infection of primary calvarial osteoblasts resulted in a significant augmentation of MMP-13 gene expression induced by LPS stimulation when compared with cells infected with control virus (*p* < 0.05) (Fig. 3).

**Figure 4:**
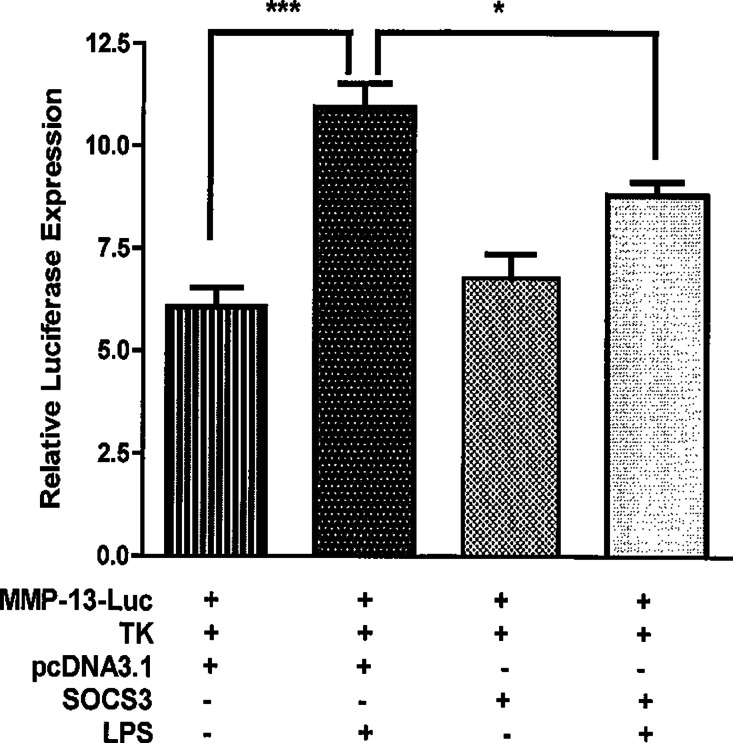
SOCS3 inhibits the activities of MMP-13 promoter-luciferase induced by LPS. MC3T3-E1 cells were transiently transfected with 0.5 µg of DNA comprised of a mouse MMP-13 luciferase gene, thymidine kinase-luciferase gene, and either SOCS3 expression plasmid or control vector. 24 h after transfection, the cells were incubated with or without 100 ng LPS/ml for 4 h. Cell lysates were used to perform luciferase activity assay. Luminometer values were normalized for expression from a co-transfected thymidine kinase reporter gene. The data were expressed as means ± S. E. M., *n* = 3. A representative experiment of a total of two similar experiments is shown. ∗ and ∗∗∗ indicate a statistically significant difference- *p* < 0.05 and *p* < 0.001, respectively.

To further test the finding that SOCS3 decreases LPS-induced MMP-13 expression in osteoblasts, we performed a transient transfection assay using MMP-13-promoter luciferase reporter and SOCS3-expressing plasmid. Twenty-four hours after transfection, MC3T3-E1 cells were treated with or without LPS for 4 h before harvesting the cells. Consistent with the results from qRT-PCR (Figs. 2 and 3), LPS stimulation in the absence of SOCS3-expressing plasmid led to a significant increase in luciferase activity compared with untreated MC3T3-E1 cells (Fig. 4, *p* < 0.001). The data also indicate that LPS treatment of SOCS3-transfected MC3T3-E1 cells suppressed luciferase activity over the reporter alone (Fig. 4, *p* < 0.05). Furthermore, the group of cells with no LPS treatment that were transfected with SOCS3-expressing plasmid demonstrated a similar level of luciferase activity with that of the control group (Fig. 4), suggesting that SOCS3 works only in conjugation with LPS stimulation.

### SOCS3 inhibits LPS-induced MAP kinase activity in osteoblasts

**Figure 5:**
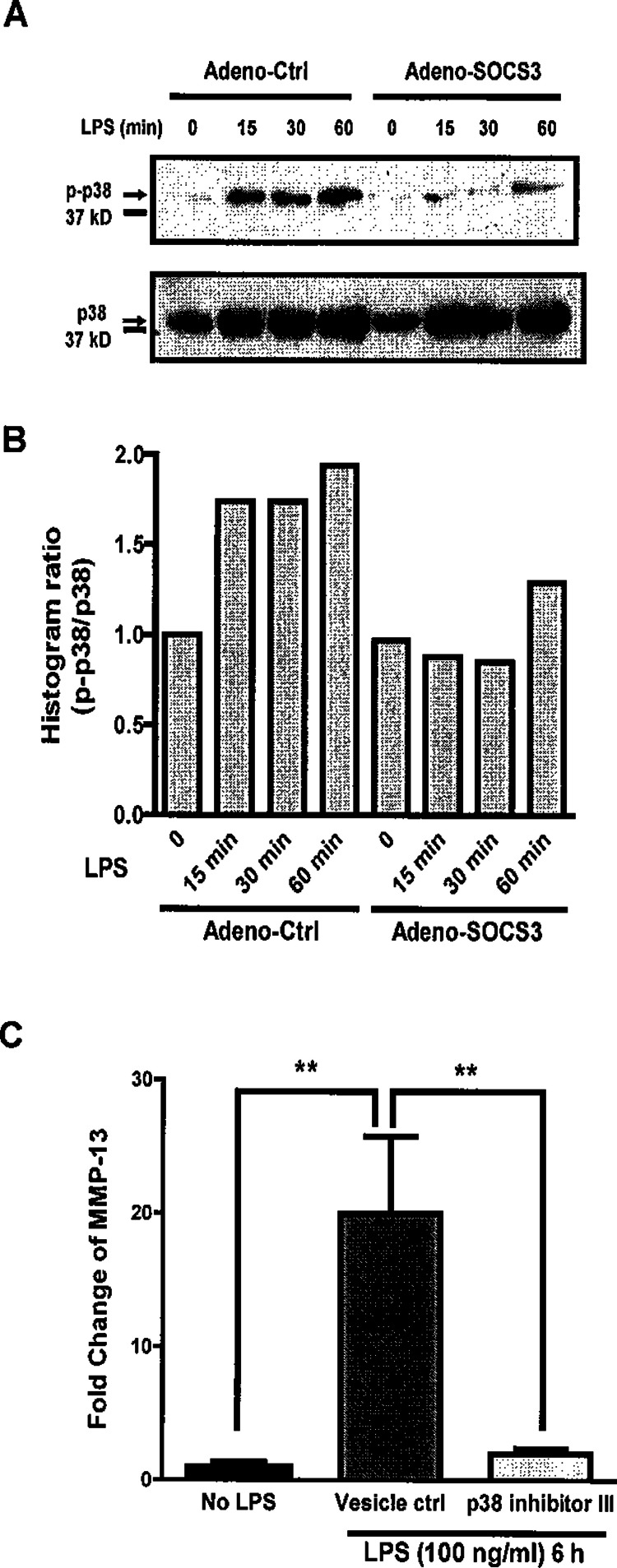
SOCS3 inhibits LPS-induced MMP-13 expression through p38 MAP Kinase pathway in MC3T3-E1 cells. (A) MC3T3-E1 cells were infected with Adeno-Ctrl or Adeno-SOCS3 at 100 X MOI. 48 h later, the cells were stimulated either with or without 100 ng LPS/ml for the indicated time periods. Total proteins were subjected to Western blot by using rabbit anti-phospho-p38 (p-p38) antibody, rabbit anti-p38 antibody (p38), respectively. (B) Western blot were digitized and analyzed. p-p38 expression was normalized to p38 expression and the data are presented as a ratio. (C) MC3T3-E1 cells were infected with Adeno-Ctrl or Adeno-SOCS3 at 100 X MOI. 48 h later, the cells were stimulated either with or without 100 ng LPS/ml in the presence or absence of p38 MAP Kinase Inhibitor VII (10 µM) or vehicle control (DMSO) for 6 h. Total cellular RNA was isolated for real time PCR with primers for MMP-13 and GAPDH, respectively. Data are presented as mean ± S. E. M., *n* = 6. A representative experiment of a total of two similar experiments is shown. ∗∗ indicate a statistically significant difference-*p* < 0.01.

We next evaluated the potential mechanism (signal pathway) by which SOCS3 suppressed MMP-13 expression in osteoblasts. All the the mitogen-activated protein kinase (MAPK) pathways (ERK1/2, p38, and JNK) have been shown to be involved in MMP-13 expression in response to various stimuli and stress (Nakai et al., 2012; Onodera et al., 2002; Rossa et al., 2007; Zhang et al., 2012). However, the MAPK pathways which are critical in the LPS-induced MMP-13 gene regulation remain largely unknown. A previous study demonstrated that MMP-13 mRNA induction in murine periodontal ligament fibroblasts by LPS was significantly reduced by inhibition of p38 MAPK, suggesting that LPS-induced MMP-13 is regulated by p38 signaling (Rossa et al., 2007). Based on this result, we performed western blot analysis to determine whether SOCS3 may inhibit MMP-13 expression via suppressing p38 MAPK activity in osteoblasts. As shown in Fig. 5A, LPS induced p38 phosphorylation in MC3T3-E1 cells over the time course of treatment. Notably, SOCS3 significantly inhibited LPS-induced p38 phosphorylation, but has no major impact on p38 expression (Fig. 5A). Interestingly, SOCS3 had no effect on LPS-induced ERK1/2 phoshorylation in osteoblasts (data not shown).

We next determined the influence of the p38 phosphorylation on LPS-induced MMP-13 expression by using specific pharmacological inhibitors for p38 MAPK. As shown in Fig. 5B, p38 MAPK inhibitor VIII markedly suppressed osteoblast MMP-13 gene expression induced by LPS. Taken together, these results suggest that p38 MAPK is a critical signal pathway in LPS-induced MMP-13 gene expression in osteoblasts, which is inhibited by SOCS3.

## Discussion

Relationships between inflammation and bone metabolism have been established in various clinical settings and animal models of inflammatory disease (Hardy & Cooper, 2009). In particular, inflammatory processes surrounding the skeleton affect the remodeling of nearby bone tissue, often causing an increase in bone resorption by osteoclasts (Lerner, 2006). At present, the underlying mechanisms and signaling pathways by which inflammation impacts bone architecture remain poorly understood. Moreover, little is known regarding the downstream activities in osteoblasts following bacterial infection. LPS is a component of the outer membrane of gram-negative bacteria and elicits potent immune responses in animals (Gao et al., 2007; Kadono et al., 1999; Orcel et al., 1993). LPS stimulation constitutes the initial step in a cascade of events that can lead to diseases caused by gram-negative bacterial infections, such as sepsis (Chow et al., 1999). It has been reported that LPS modulates bone resorption by regulating the activities of both osteoblasts and osteoclasts (Xing et al., 2011). Specifically, LPS promotes pre-osteoclast activity via binding to toll-like receptor 4 (TLR4). Differentiated osteoblasts also express functional TLR4, which seems to play an important role in the pathogenesis of LPS-induced bone disorders (Cassatella et al., 1999; Inada et al., 2006; Kikuchi et al., 2001; Takami et al., 2002). A recent study showed that optimal osteoclastogenesis *in vitro* requires TLR4 expression in both bone marrow monocytes and osteoblasts (Zhuang et al., 2007), suggesting that bacterial stimuli such as LPS work explicitly through TLR4. However, detailed signaling pathways following LPS binding to TLR4 on osteoblasts have yet to be elucidated. While LPS signaling in macrophages and osteoclasts have been extensively studied, its exact role in osteoblasts remains largely unknown.

### LPS stimulation of MMP-13 transcriptional expression in osteoblasts

In this study, we investigated the impact of LPS on the transcriptional activation of MMP-13, a central regulator of bone resorption, in osteoblasts. As shown in Figs. 1–4, both primary murine calvariae osteoblasts and mouse osteoblast-like cells, MC3T3-E1, exhibit significant increases in MMP-13 mRNA expression upon stimulation with *E. coli* LPS. This is the first report showing *E. coli* LPS induction of MMP-13 expression in mouse osteoblasts thus far. During the reviewing of this manuscript, Barnes et al.published a report showing that triclosan inhibits LPS-induced MMP-13 expression in a rat osteoblastic osteosarcoma cell line (Barnes et al., 2013). However, the source of LPS used in this study is not known. The up-regulatory actions of LPS on MMP-13, an enzyme exclusively present in fetal skeletal development and in certain diseases involving bone resorption, suggests MMP-13 to be a key bone-resorbing perpetrator expressed by osteoblasts in inflammatory bone diseases. Taken together with previously reported LPS-induction of inflammatory mediators in osteoblasts (Nair et al., 1996), this finding strengthens the understanding of osteoblast-mediated immune response conveyed in inflammatory bone diseases.

### Role of SOCS3 in LPS-induced MMP-13 expression

The role of SOCS3 in inflammation is complex and has been a popular topic in both innate and adaptive immunity fields during the past decade. Research surrounding SOCS3 has also been controversial, as both pro- and anti-inflammatory functions of SOCS3 have been demonstrated. For example, SOCS3 plays a critical role in preventing interferon-γ (IFN-γ)-like responses in cells stimulated by IL-6 (Croker et al., 2003; Lang et al., 2003), which promotes both acute and chronic inflammation in the absence of SOCS3 *in vivo* (Croker et al., 2012). Conversely, mice lacking SOCS3 in neutrophils and macrophages are resistant to LPS-induced shock, indicating that SOCS3 may function as a pro-inflammatory mediator by suppressing IL-6 signaling, interfering with its ability to inhibit LPS signaling (Seki et al., 2003; Yasukawa et al., 2003). This conclusion is supported by a recent report showing that SOCS3 promotes TLR4 response in macrophages by feedback inhibiting TGFβ1 signaling (Liu et al., 2008). Thus, understanding the roles of SOCS3 in various diseases is critical to revealing insights into signaling pathways that can be manipulated in potential therapeutic approaches.

SOCS3 is expressed in all major bone cells including osteoclasts, chondrocytes, and osteoblasts (Ohishi et al., 2005; van de Loo et al., 2012; Yan et al., 2010). Interestingly, a recent study demonstrated that SOCS3 is highly expressed in human arthritic chondrocytes and affects the production of nitric oxide (NO) and proteoglycans (van de Loo et al., 2012). In addition, this study shows that there is a strong positive correlation between SOCS3 expression and that of genes that are putatively involved in the arthritic process including MMP13. Thus, they propose that SOCS3 could play a central role in the pathophysiology of joint diseases by deregulating chondrocyte function. However, investigation of the SOCS3 function in the bone remodeling system, specifically in osteoblasts, is still in its early stages. Our current study shows that over-expression of SOCS3 drastically down-regulates LPS-induced MMP-13 gene expression in both primary murine calvariae osteoblasts and MC3T3-E1 cells (Figs. 2 and 4). Furthermore, SOCS3 knockdown leads to a significant increase of LPS-induced MMP-13 gene expression in MC3T3-E1 cells (Fig. 3). These findings strengthen the characterization of SOCS3 as an anti-inflammatory signaling molecule in osteoblast-mediated immune responses.

As shown in Fig. 1A, endogenous levels of SOCS3 decreases continuously following *E. coli* LPS stimulation while MMP-13 expression significantly increases at 6 and 24 h following *E. coli* LPS treatment. Thus, in order to effectively suppress *E. coli* LPS-induced MMP-13 transcription, an adequate expression of SOCS3 may be necessary. Additionally, other unknown molecules may be involved in the down-regulation of MMP-13 expression at 48 h after *E. coli* LPS treatment since SOCS3 expression is also very low at this time point (data not shown).

MMP-13 expression can be regulated by MAPK in response to various stimuli and in different cells (Nakai et al., 2012; Onodera et al., 2002; Rossa et al., 2007; Zhang et al., 2012). However, how SOCS3 regulates MAPK in osteoblast is not known. Using p38 MAP kinase inhibitor, a previous study shows that LPS-stimulated MMP-13 mRNA induction was significantly reduced by inhibition of p38 MAP kinase in murine periodontal ligament fibroblasts. Thus, our results that LPS treatment led to the phosphorylation of p38 MAP kinase is consistent with this report (Rossa et al., 2007). Importantly, our results suggest that SOCS3 plays a critical role in LPS-induced MMP-13 gene expression in osteoblast by regulating p38 MAPK pathway. However, the molecular details of SOCS3 regulation of signaling pathways downstream of TLR4 in osteoblasts remain to be determined.

## Conclusions

We show that LPS significantly increases MMP-13 mRNA expression in both primary murine calvariae osteoblasts and osteoblast-like cells, MC3T3-E1. These findings together with related bone inflammation literature (Nair et al., 1996; Yan et al., 2010), enhance the connection between the bone remodeling process and inflammation. In addition, we identify a novel regulatory role of SOCS3 in osteoblast-mediated inflammatory responses in MC3T3-E1 cells: through over-expression and knockdown of SOCS3 protein, we demonstrate, for the first time, that SOCS3 suppresses MMP-13 transcriptional activation following LPS stimulation in osteoblasts. Exploring the underlying mechanisms and signaling pathways regulating SOCS3 expression in osteoblasts could lead to important new knowledge involving therapeutic targeting of MMP-13 in inflammation-resolving approaches.
