# Correction: A critical role for suppressors of cytokine signaling 3 in regulating LPS-induced transcriptional activation of matrix metalloproteinase-13 in osteoblasts

**Published:** 2013-05-20

This article has been corrected to reflect the correct initials of the third author. The original article incorrectly stated his name as "Bruno Herrera", however the correct name is "Bruno Schneider Herrera".

